# Simultaneous Oculomotor and Trochlear Nerve Palsy in Herpes Zoster Ophthalmicus

**DOI:** 10.7759/cureus.30755

**Published:** 2022-10-27

**Authors:** Kah L Low, Masnon Nurul-Ain, Jemaima Che Hamzah, Wan-Hazabbah Wan Hitam

**Affiliations:** 1 Ophthalmology, University Kebangsaan Malaysia, Kuala Lumpur, MYS; 2 Ophthalmology, Kuala Lumpur Hospital, Kuala Lumpur, MYS; 3 Ophthalmology, School of Medical Sciences, University Sains Malaysia, Kota Bharu, MYS

**Keywords:** herpes zoster ophthalmicus, cranial neuropathies, ophthalmoplegia, ptosis, keratouveitis

## Abstract

Simultaneous oculomotor and trochlear nerve palsy in Herpes Zoster Ophthalmicus (HZO) is rare. We report a 78-year-old lady who presented with right ptosis while receiving treatment for HZO keratouveitis. Examination showed crusted vesicular lesions on the right V_1_ dermatome with right complete ptosis and limited right eye extraocular movement on adduction, supraduction, infraduction, and intorsion. There was mild right eye anterior chamber inflammation, while bilateral fundus examinations were normal. Other neurological and systemic examinations were unremarkable. Neuroimaging was also normal. Corticosteroids treatment was started, in addition to oral acyclovir, given for a total course of six weeks. Her ocular motor cranial neuropathy (OMCN) was partially resolved during the follow-up at one year. OMCN can be an isolated complication of HZO, or it may be associated with other neurological complications. Although recovery for OMCN is anticipated, the patient should be closely monitored for the possibility of developing other devastating neurological complications.

## Introduction

Herpes zoster (HZ) is classically presented as a painful, blistering dermatomal rash. The primary affected demographic is above 50 years of age [1]. Ocular motor cranial neuropathy (OMCN) is a rare presentation that accounts for only 1 to 3% of HZ [2]. It can occur as an isolated consequence of Herpes Zoster Ophthalmicus (HZO) or accompanying other neurological complications. Simultaneous unilateral third and fourth cranial nerve palsy in HZO is uncommon. We report and discuss the clinical presentation, management, and possible neurological complications of OMCN in HZO cases.

## Case presentation

A 78-year-old lady with underlying hypertension presented with right eye redness and discomfort after five days of having painful vesicular skin rashes over the right forehead, periocular skin, and tip of the nose. She was diagnosed with right eye Herpes Zoster Ophthalmicus (HZO) keratouveitis as there was evidence of punctate keratitis, keratic precipitate, endothelial striae, and inflammatory cells of 1+ in the anterior chamber. She was started on topical acyclovir, corticosteroids, and cycloplegic, as well as oral acyclovir. After one week, she presented with an acute onset of right ptosis. She did not have a fever, headache, neck stiffness, nausea, vomiting, or limb weakness.

On examination, visual acuity was 6/9 bilaterally. Skin lesions on the right forehead, periocular area, and tip of the nose, which is known as the Hutchinson sign, were scarring up. She had right complete ptosis, with restrictions of ocular movement in adduction, supraduction, infraduction, and intorsion. Cornea sensation was reduced on the right side. Anterior chamber showed occasional cells. The pupil was 5mm round and dilated due to a cycloplegic agent. Optic nerve functions were intact, and fundus examinations were unremarkable. She was alert and afebrile. The remainder of the systemic and neurological examinations were normal.

**Figure 1:**
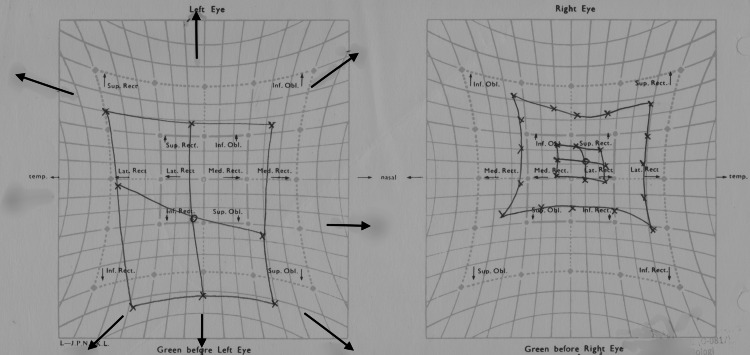
Hess chart revealing under actions of right medial, superior, and inferior rectus, inferior oblique, and superior oblique muscle

Hess chart showed under action of the right medial rectus, superior rectus, inferior rectus, inferior oblique, and superior oblique muscles consistent with right oculomotor and trochlear nerve palsy (Figure 1).

Normal laboratory findings were demonstrated through complete blood count, erythrocyte sedimentation rate, and C-reactive protein. Contrast-enhanced CT (CECT) and CT angiography of the brain and orbit revealed no enhancing lesion in the brain, meninges, cavernous sinus, orbit, and orbital apex with no evidence of aneurysm or cerebral vasculitis.

**Figure 2:**
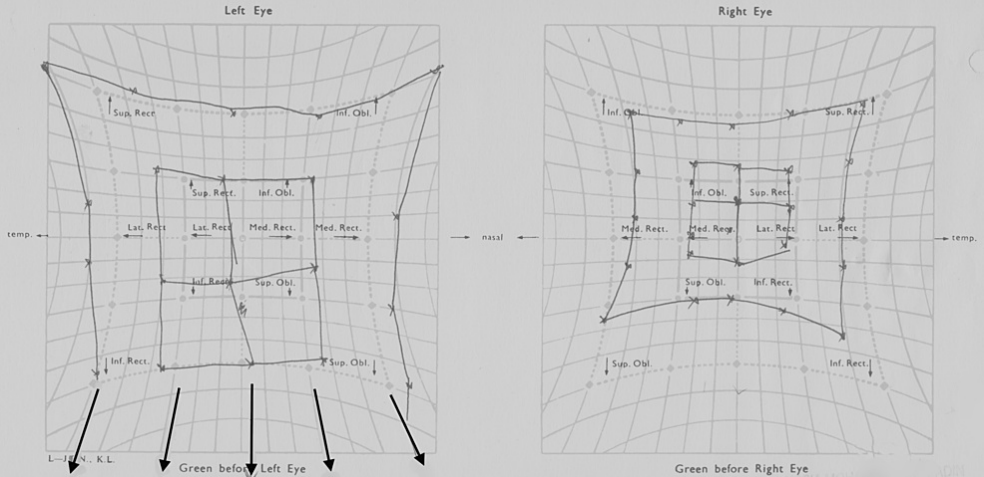
Repeated Hess chart one year later showing partial improvements of right medial, superior, and inferior rectus, inferior oblique, and superior oblique muscle

A diagnosis of right oculomotor and trochlear nerve palsy with HZO keratouveitis was made. She was initiated on oral acyclovir (800mg five times per day) and prednisolone (1mg/kg/day) with tapering doses over a six-week period without any serious side effects. Topical medications were discontinued after three months. At the one-year follow-up, her right ptosis recovered, and ocular motility had significantly improved (Figure 2).

There was no recurrence of keratouveitis, and she remained well without other neurological sequelae of HZO.

## Discussion

Ocular motor cranial neuropathy (OMCN) happens in 5-31% of HZO. It occurs more in the elderly and is mostly ipsilateral to the dermatome involved, but contralateral and bilateral cases have also been reported [1]. The most commonly involved OMCN was the oculomotor nerve (CN III) and subsequently followed by the abducens nerve (CN VI) and trochlear nerve (CN IV) [1]. Simultaneous more than one OMCN in HZO have been documented but exceptionally rare.

Reactivation of varicella-zoster virus (VZV) may occur due to advanced age, immunosuppression, and severe stressors. Several mechanisms have been postulated for the cause of OMCN in HZO. Direct cytopathic effect via sensory ramifications of the trigeminal nerve, or contiguous inflammation from the cavernous sinus or the superior orbital fissure to the ocular motor cranial nerves, has been suggested in causing OMCN. VZV may also cause myositis to the extraocular muscles, perineuritis, thrombophlebitis, and occlusive vasculitis leading to microinfarction and ischemia of the ocular motor cranial nerves [1].

OMCN typically appears later after the onset of rashes, with 75% of cases presenting after a range of two to 42 days. Our patient's experience is in accordance with this, as she presented with ptosis and ophthalmoplegia 12 days after the onset of vesicular rashes. Others reported that OMCN can be concomitant or even precede the onset of rashes, while some may develop OMCN without rashes. Concurrent anterior segment involvement has been found to occur in 90% of patients with OMCN, as evident in our patient, while 20% of the patients are also at risk of posterior segment complications. Multiple OMCN is relatively infrequent compared to isolated ocular motor palsy. To our knowledge, there were only two cases of simultaneous oculomotor and trochlear nerve palsy in HZO reported in the literature [2].

OMCN in HZO can be a presentation of meningitis or encephalitis. Haargaard et al. reported a case of ipsilateral facial palsy, encephalitis, and contralateral hemiparesis following oculomotor palsy in HZO, due to diffuse cerebral vasculitis [3]. Recently, Zhu et al. reported a case of cerebral venous thrombosis following HZO [4]. Neurological complication occurs in 5.5% of HZ, and the risk increases sharply in HZO [2]. Other known HZ neurological complications include meningorediculitis, cerebellitis, cerebrovascular accident, and myelitis with sensory, motor, and autonomic dysfunction. On the other hand, post-herpetic neuralgia (PHN) is a chronic pain syndrome and represents the commonest complicating pathology of HZ [1]. Severe PHN can result in a depressed mood, loss of weight, interrupted sleep, and inability to perform daily tasks. Fortunately, throughout the course of a year's worth of three monthly follow-ups, our patient had had none of these complications. 

Neuroimaging is recommended for HZO OMCN to determine whether it occurs in isolation or is associated with radiological evidence of other neurological complications. Ophthalmoplegia may also result from orbital lesions, which neuroimaging may reveal through signs of myositis or orbital soft tissue enhancement. It is interesting to note that some HZ cases complicated with encephalitis and meningitis may still have normal neuroimaging without any pathological signs of infection or inflammation in the brain parenchyma [5]. Hence, lumbar puncture should be done, and diagnostic confirmation using polymerase chain reaction for cerebrospinal fluid should be obtained in those cases of diagnostic uncertainty [5].

Consumption of systemic antiviral agents (acyclovir, valacyclovir, or famciclovir) within 72 hours onset of rashes has a favorable effect in improving dermatologic healing, reducing pain and the rate of complications of HZ and HZO [5]. Intravenous acyclovir is indicated in patients with other serious neurological involvement. Our patient received oral acyclovir only five days after the onset of rashes, and we prolonged the oral acyclovir for a total of six weeks. The guidelines for antiviral or corticosteroid treatment on HZO OMCN have not been formally established. Kedar et al. suggested a short course (five days) of corticosteroids for OMCN [1], while we extended and tapered the corticosteroids over a six-week period based on clinical judgment due to the patient's slow recovery. It is worth noting that the US FDA has approved the use of the recombinant zoster vaccine (RZV) to eliminate the risk of developing HZ and PHN with an efficacy of 91.3% and 88.8%, respectively [1].

Twenty-five percent of HZO has a tendency to recur in five years [1]. However, the prognosis of HZO OMCN is generally good, with improvement seen within two weeks to one and a half years. Very few cases may need squint surgery due to poor recovery [1].

## Conclusions

HZO may present with delayed complications such as OMCN or other serious neurological sequelae. Early initiation of antiviral therapy is recommended in HZ. Vaccination is ideal for preventing serious ocular and neurological complications of HZ. On the other hand, it is crucial to closely monitor and conduct regular follow up of HZO patients. Treatment for HZO OMCN has not been fully developed due to its rarity. HZO OMCN typically has a fair prognosis and recovery.
